# What is the clinical significance of ‘heteroresistance’ in nonfermenting Gram-negative strains?

**DOI:** 10.1097/QCO.0000000000000964

**Published:** 2023-09-20

**Authors:** Giusy Tiseo, Valentina Galfo, Marco Falcone

**Affiliations:** Infectious Diseases Unit, Department of Clinical and Experimental Medicine, Azienda Ospedaliero Universitaria Pisana, University of Pisa, Pisa, Italy

**Keywords:** *Acinetobacter baumannii*, cefiderocol, Gram-negative bacilli, heteroresistance, multidrug resistance

## Abstract

**Purpose of review:**

The aim of this study was to discuss the potential clinical significance of heteroresistance in nonfermenting Gram-negative bacilli (GNB).

**Recent findings:**

Recently, heteroresistance has been considered potentially responsible for clinical failure in *Acinetobacter baumannii* infections. This raised a scientific debate, still open, about the potential clinical significance of heteroresistance in nonfermenting GNB.

**Summary:**

We reviewed the literature of last 20 years and found a limited number of studies evaluating the relationship between heteroresistance and clinical outcome in nonfermenting GNB. Unlike Gram-positive bacteria, heteroresistance is reported in a significant proportion of nonfermenting GNB with some studies describing it in all tested strains and for several antibiotics (including tigecycline, carbapenems, levofloxacin, cefiderocol, colistin). One important issue is the need for validated detection method since the population analysis profile test, that is considered the gold standard, requires high costs and time. Studies evaluating the correlation between heteroresistance and clinical outcome are contrasting and have several limitations. Although in-vitro detection of heteroresistance in nonfermenting GNB has not been associated with in-vivo treatment failure, its presence may suggest to prefer combination regimens instead monotherapy when treating infections by nonfermenters. Further studies are needed to clarify the clinical significance of heteroresistance.

## INTRODUCTION

Antibiotic resistance is a global threat to human health. Patients with infections by multidrug-resistant (MDR) Gram-negative bacilli (GNB) have an increased risk of poor outcome and mortality attributable to specific cause [1^▪^,^2^,^3^]. Over the past decades, the understanding of the mechanisms underlying antibiotic resistance has significantly increased [4,5]. Advances in molecular biology allowed the identification of complex resistance mechanisms able to confer a phenotype of resistance [6–8]. However, some phenomena, such as heteroresistance, remain difficult to be understood and their impact on clinical practice is still debated.

Heteroresistance is a phenotype in which a bacterial isolate contains subpopulations with reduced antibiotic susceptibility compared to the main population [9]. These populations pose a clinical concern, as they could become prevalent during or after antibiotic exposure. Heteroresistance was first described in the 1940 s when streptomycin resistance in type B *Haemophilus influenzae* emerged during exposure to this antibiotic leading to clinical failure [10]. Heteroresistance has been extensively studied for Gram-positive bacteria, in particular for heterogeneous vancomycin intermediate *Staphylococcus aureus* (hVISA). A recent meta-analysis showed a prevalence of 6% of hVISA among methicillin-resistant *Staphylococcus aureus* (MRSA) isolates [11,12]. Of note, there is heterogeneity in detection methods and controversial findings from available studies. Several clinical studies evaluated the association between hVISA and clinical failure, including persistent bacteraemia and treatment failure, but data on mortality are inconclusive [12].

Currently, heteroresistance among nonfermenting GNB drew the attention of scientific community because a debate about its potential role in explaining the clinical failure in some trials recently emerged [13^▪▪^,^14^]. Compared to hVISA, data about heteroresistance in nonfermenting GNB are limited and poorly evaluated.

In this review, we critically assess the published literature on heteroresistance in nonfermenting GNB. We underline that this is not a microbiological review, and we do not examine the specific mechanisms conferring heteroresistance. Conversely, we provide a clinical point of view and highlight the potential relationship between heteroresistance and clinical outcome of patients with infections by nonfermenting GNB. 

**Box 1:**
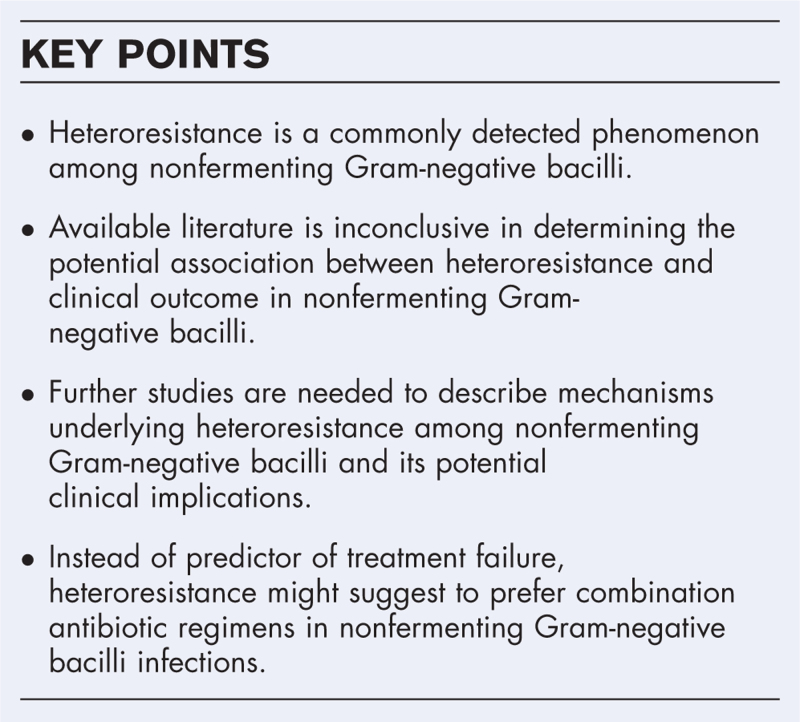
no caption available

## DEFINITION OF HETERORESISTANCE

In general, heteroresistance is the presence of a heterogeneous population of bacteria with one subpopulation or several subpopulations that exhibit increased levels of antibiotic resistance compared with the main population [15]. However, this definition is incomplete lacking information of resistant subpopulations. The only type of heteroresistance with a clear consensus definition is the hVISA [defined as a *S. aureus* isolate susceptible to vancomycin (MIC 2 mg/l) but with minority populations (>10^–6^ cells] growing on vancomycin >2 mg/l by population analysis profile (PAP) investigation]. For other pathogens, especially for GNB, there is no global consensus on the definition of heteroresistance.

**FIGURE 1:**
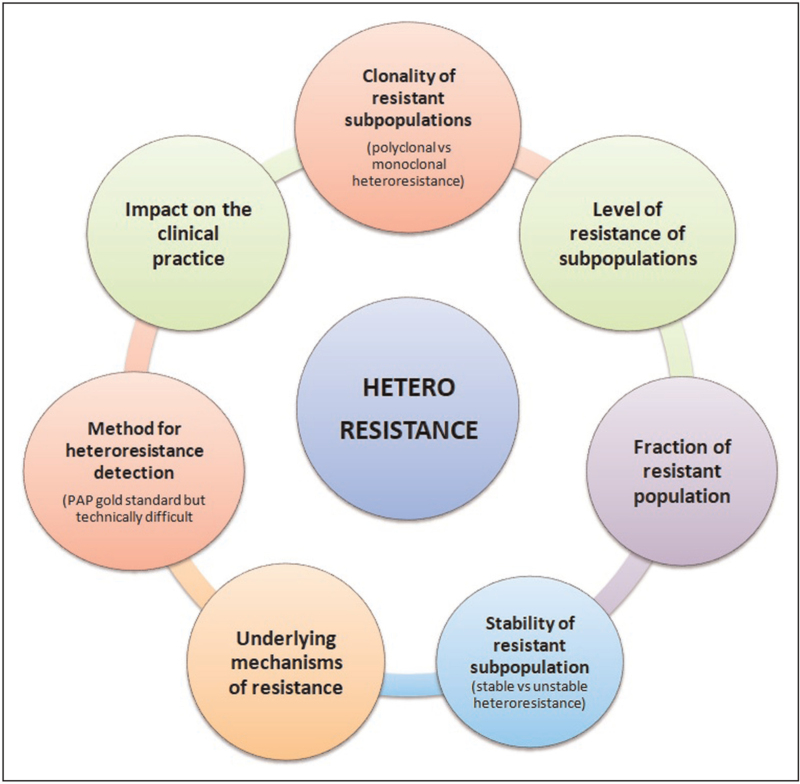
Factors to consider when defining antibiotic heteroresistance.

Figure 1 summarizes factors to be considered in the description of heteroresistance. The first factor is the clonality of resistant subpopulation. Both polyclonal and monoclonal heteroresistance may occur. In the first case (*polyclonal heteroresistance*), heteroresistance results from mixed infections or from rare resistant mutants that slowly increase in proportion during antibiotic treatment in a population of susceptible bacteria [9]. In the second case (*monoclonal heteroresistance*), heteroresistance may be generated from a single clone that differentiates into two populations (susceptible and resistant) at a high frequency in the absence of antibiotic pressure [9]. The second factor is the resistance level of the resistant subpopulations. Defining a minimal fold of increase in resistance for the subpopulation (defined as x-fold higher than the MIC of the main population) is important since it ensures that heteroresistance does not describe subpopulations with limited increases in MIC. Furthermore, heteroresistance may be classified as stable or unstable. The *unstable heteroresistance* occurs when the resistant subpopulations can revert to susceptibility in the absence of antibiotic pressure. Moreover, different mechanisms, including point mutations, insertion sequences and small deletions, may be involved in the develop of antibiotic heteroresistance and should be investigated.

Finally, several detection methods, including the PAP test, the microdilution PAP test, the PAP–AUC, broth microdilution, E-test and disc diffusion, are used [16].

## EVIDENCES FROM THE LITERATURE

**FIGURE 2:**
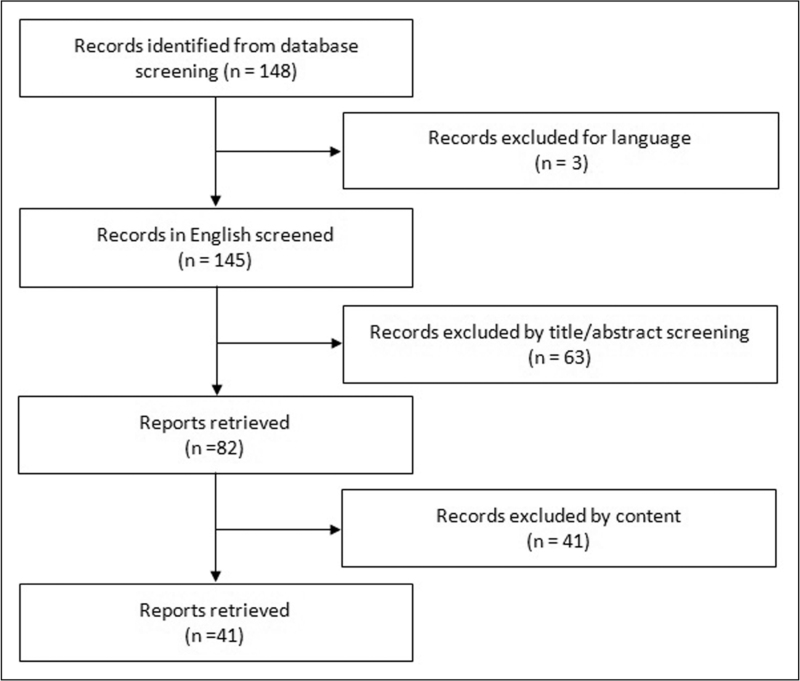
Systematic review of studies evaluating the correlation between heteroresistance and clinical outcome in nonfermenting Gram-negative bacilli.

To better summarize the available evidences about the relationship between heteroresistance and clinical outcome in infections by nonfermenting GNB, we performed a systematic review of the literature. A PubMed search (January 2003--July 2023) with the following searching terms (heteroresistance) AND (Gram negative OR *Stenotrophomonas* OR *Acinetobacter* OR *Pseudomonas*) was carried out. Narrative reviews were analysed to search for other relevant studies [15,17]. The flow diagram of the literature review is shown in Fig. 2. We identified a total of 148 studies. After screening by title/abstract and full text review, 42 studies evaluating heteroresistance in nonfermenting GNB were included. A summary of the main findings of the selected studies is reported in Table 1[13^▪▪^,^14^,^18^^▪▪^,^19^–^56^].

**Table 1:** Summary of studies evaluating the correlation between heteroresistance and clinical outcome in nonfermenting Gram-negative bacilli (last 15 years)

Ref.	Pathogen	Antibiotic	Type of study	Detection method	% of HR	Impact on clinical outcome
Choby *et al*. [13^▪▪^]	*A. baumannii*	Cefiderocol	Microbiological	PAP	59%	The authors hypothesized that clinical failure of CREDIBLE-CR is due to cefiderocol-HR among CRAB isolates.
Choby *et al*. [14]	*A. baumannii*	Cefiderocol	Microbiological	PAP	20%	The authors hypothesized that the better outcome of patients who received cefiderocol in the APEKS-NP trial compared to CREDIBLE-CR is due to the lower cefiderocol-HR in susceptible GNB (APEKS-NP).
Stracquadanio *et al*. [18^▪▪^]	*A. baumannii*	Cefiderocol	Microbiological	PAP	80%	--
Jo *et al*. [19]	*A. baumannii*	Colistin, tigecycline	Microbiological	PAP	100%	--
Jo *et al*. [20]	*A. baumannii*	Tigecycline	Microbiological	PAP	56%	--
Kon *et al*. [21]	*A. baumannii*	Colistin	Clinical	PAP	67.1%	No significant increase in 14-day mortality or 14-day clinical failure, exception made for bacteraemia.
Kon *et al*. [22]	*A. baumannii*	Colistin	Microbiological	PAP	100%	--
Karakonstantis *et al*. [23]	*A. baumannii*	Colistin	Systematic review and meta-analysis	PAP	33%	A single included study evaluating correlation between HR and clinical outcome
Hong *et al*. [24]	*A. baumannii*	Colistin	Microbiological	PAP	50%	--
Çağlan *et al*. [45]	*A. baumannii*	Colistin	Microbiological	PAP	21%	
Ezadi *et al*. [44]	*A. baumannii*	Colistin	Microbiological	PAP	21%	
Srinivas *et al*. [25]	*A. baumannii*	Colistin	Clinical	Mueller-Hinton agar plates	83%	No difference in 30-day mortality between susceptible and HR strains.
Choi *et al*. [26]	*A. baumannii*	Colistin	A clinical case with multiple isolates	PAP	84.2% of tested isolates from the same patient	Clinical failure of the case
Juhász *et al*. [43]	*A. baumannii* *P. aeruginosa*	Colistin	Microbiological	Mueller–Hinton agar plates	20% *A. baumannii* 27% *P. aeruginosa*	--
Rao *et al*. [27]	*A. baumannii*	Colistin	Microbiological	PAP	100%	--
Rodriguez *et al*. [42]	*A. baumannii*	Colistin	Microbiological	PAP	95%	--
Barin *et al*. [41]	*A. baumannii*	Colistin	Microbiological	PAP	90%	--
Cai *et al*. [28]	*A. baumannii*	Colistin	Systematic review	Not reported	19–100%	Combination therapy seems to be a strategic choice for HR isolates
Rodriguez *et al*. [29]	*A. baumannii*	Colistin	Microbiological	PAP	86%	--
Hung *et al*. [36]	*A. baumannii*	Cephalosporin	Case series	DD, Etest, PAP		Previous exposure to cephalosporins may have selected HR-population
Fernandez-Cuenca *et al*. [37]	*A. baumannii*	Carbapenem	Microbiological	DD	IMI: 20% MER: 25%	
Vidaillac *et al*. [40]	*A. baumannii* *P. aeruginosa*	Colistin	Microbiological	PAP	100%	
Lee *et al*. [35]	*A. baumannii*	Imipenem	Clinical	DD, Etest	58%	No difference in mortality, but higher exposure to IMI in controls
Rodriguez *et al*. [30]	*A. baumannii*	Colistin	Microbiological	Time killing	43%	.
Rodriguez *et al*. [31]	*A. baumannii*	Colistin	Microbiological	PAP	46%	
Savini *et al*. [33]	*A.baumannii*	AMP/SUL	Microbiological	DD	25%	
Ikonomidis A *et al*. [34]	*A.baumannii*	Carbapenem	Microbiologica	PAP	100% of susceptible isolates	
Yau *et al*. [39]	*A. baumannii*	Colistin	Microbiological	PAP	23%	
Hawley *et al*. [32]	*A. baumannii*	Colistin	Microbiological	Mueller-Hinton agar	100%	
Li *et al*. [38]	*baumannii*	Colistin	Microbiological	PAP	94%	
Monogue *et al*. [56]	*P. aeruginosa*	C/T	Microbiological	PAP	23%	
Howard-Anderson *et al*. [47]	*P. aeruginosa*	Colistin	Clinical	PAP	6--26%	No difference in 90-day mortality between susceptible and HR strains
Li *et al*. [48]	*P. aeruginosa*	Levofloxacin	Microbiological	PAP	66.7%	
Jia *et al*. [49]	*P. aeruginosa*	Cefepime	Clinical	DD or Etest then PAP	57%	Treatment failure higher in infections by HR isolates.
He *et al*. [50]	*P. aeruginosa*	Carbapenems	Clinical	Disk diffusion, PAP in 9 isolates	IMI: 54% MEM: 72% IMI+MEM: 42%	No difference of mortality between HR and susceptible strains.
Hermes *et al*. [55]	*P. aeruginosa*	Colistin	Microbiological	PAP	4.2%	
Bergen *et al*. [51]	*P. aeruginosa*	Colistin	Microbiological	PAP	66.7%	
Pournaras S *et al*. [52]	*P. aeruginosa*	TZP	Case report	DD, PAP	A single isolate before therapy start	Favourable outcome reported in the first case of HR strain treated with TZP
Ikonomidis A *et al*. [54]	*P. aeruginosa*	Carbapenems	Microbiological	PAP	100%	
Pournaras *et al*. [53]	*P. aeruginosa*	Carbapenems	Microbiological	DD, PAP	27.5%	
Walsh *et al*. [47]	*P. aeruginosa*	Fosfomycin	Microbiological	PAP	100%	

AMP/SUL, ampicillin/sulbactam; C/T, ceftolozane/tazobactam; DD, disc diffusion; HR, heteroresistance; IMI, imipenem; MEM, meropenem; PAP, population analysis profile; TZP, piperacillin/tazobactam.

We highlight some important findings from the literature review:

(1) heteroresistance is commonly detected among nonfermenting GNB for several antibiotics, including polymyxins, β-lactams (carbapenems, piperacillin/tazobactam, cefiderocol), tigecycline, fosfomycin;
(2)
heteroresistance has been studied mainly for *A. baumannii* and *P. aeruginosa*, while no study was performed in *S. maltophilia;*
(3) the majority of studies are microbiological experiments, while clinical studies evaluating the correlation between heteroresistance and clinical outcome of patients are limited (five for *A. baumannii* and four for *P. aeruginosa*)
(4) there is heterogeneity in the detection methods.

These observations highlight that the knowledge of heteroresistance in nonfermenting GNB is less consolidated compared to Gram-positive bacteria. Data are heterogeneous and the phenomenon is so common for all tested antibiotics that it is difficult to evaluate its potential clinical role.

## HETERORESISTANCE IN *ACINETOBACTER BAUMANNII*

Heteroresistance in *A. baumannii* is commonly detected for several antibiotics, including colistin, tygecicline, cefiderocol and ampicillin/sulbactam (Table 1).

Colistin-heteroresistance is the most common described type of heteroresistance in this pathogen. A meta-analysis including 15 studies showed an overall prevalence of colistin-heteroresistance among *A. baumannii* isolates of 33% [23]. Only one study on 24 patients evaluated the correlation of colistin-heteroresistance with clinical outcome and showed no differences in clinical cure and mortality between heteroresistant and homogeneously susceptible *A. baumannii* infections [23]. More recently, Kon *et al*. [21] investigated the prevalence of colistin-heteroresistance detected using the PAP and its evolution into full resistance among 173 clinical carbapenem-resistant *A. baumannii* (CRAB) isolates. Colistin-heteroresistance was detected in the 67.1% of CRAB isolates and the 80.2% of heteroresistant strains evolved into full resistance over time [21]. Of importance, in this study, colistin-heteroresistance was not significantly associated with 14-day mortality, with a trend for increased mortality observed only in patients with bacteraemia [21].

Heteroresistance in CRAB has been also detected for cefiderocol. Recently, some observational studies showed promising results in patients with CRAB infections treated with cefiderocol compared to colistin-containing regimens [57,58^▪▪^]. However, the CREDIBLE-CR trial showed higher clinical failure in patients treated with cefiderocol compared to those who received the best available therapy (BAT) [59]. Choby *et al*. [13^▪▪^,^14^] advanced the hypothesis that cefiderocol-heteroresistance may be the reason of the high clinical failure occurring in patients treated with cefiderocol. To support this hypothesis, they calculated the prevalence of cefiderocol-heteroresistance using the PAP test in CRAB isolates from the Georgia Emerging Infections Program (USA) [13^▪▪^]. They found that 59.3% of CRAB isolates showed cefiderocol-heteroresistance and postulated that heteroresistance might have contributed to cefiderocol treatment failure in the CREDIBLE-CR study [13^▪▪^]. Moreover, to explain the discrepancy of mortality rates between the CREDIBLE-CR trial and the APEKS-NP trial (including carbapenem susceptible GNB) [60], in a further experiment, they calculated the prevalence of cefiderocol-heteroresistance among carbapenem susceptible GNB from the same Georgia cohort [14]. They observed lower rates of cefiderocol-heteroresistance in carbapebem-susceptible GNB compared to those detected in the previous study (11% in fully susceptible and 44% in cephalosporin-resistant isolates), postulating that clinical failure of cefiderocol depends on the different rates of heteroresistance [14].

The debate is still open for several reasons [61]. First, data of cefiderocol-heteroresistance in CRAB come from a cohort different from CREDIBLE-CR (Georgia surveillance). Second, despite the high prevalence of colistin-heteroresistance among CRAB, heteroresistance rate and its correlation with clinical outcome in the BAT group have not been considered. Finally, there are other relevant factors that may have contributed to the increased mortality observed in the cefiderocol-treated patients of the trial, who had more frequently septic shock and were more commonly cared for in intensive care unit compared to BAT. A recent microbiological study elucidated important characteristics of cefiderocol-heteroresistant in CRAB isolates [18^▪▪^]: first, high rates of cefiderocol-heteroresistance after exposure to this drug was detected (heteroresistance detected in eight out of 10 isolates using the PAP method); second, small colonies immediately turned back to their original shape once the drug was removed, suggesting that cefiderocol-heteroresistance is unstable; third, since the strains harboured multiple classes of b-lactamase resistance genes, the addition of β-lactamase inhibitors, such as ceftazidime/avibactam, to cefiderocol may restore its activity [18^▪▪^]. Heteroresistance has been also detected among *A. baumannii* isolates for other antibiotics, including tigecycline (up to 56%) [18^▪▪^] and ampicillin/sulbactam (25% in a single study) [33].

## HETERORESISTANCE IN *PSEUDOMONAS AERUGINOSA*

In *P. aeruginosa*, heteroresistance has been reported in different studies [47–56], and its role in treatment failure was reported in a large retrospective cohort, where imipenem and meropenem heteroresistance was detected in 54 and 73% of the isolates, respectively [50]. Heteroresistance rates for colistin and levofloxacin are higher than 60% [46–48], while a single clinical case of a patients with infection by *P. aeruginosa* with heteroresistance to piperacillin/tazobactam that no lead to clinical failure has been reported [52]. Recently, ceftolozane/tazobactam-heteroresistance has been reported, with a prevalence of 23% [56]. Available studies did not demonstrate that heteroresistance in *P. aeruginosa* may increase mortality.

## EXPERT OPINION ON CLINICAL IMPLICATIONS OF HETERORESISTANCE IN NONFERMENTING GRAM-NEGATIVE BACILLI

Current available data about the role of heteroresistance among nonfermenting GNB are limited and do not clearly demonstrate its potential clinical role. Anyway, the literature review suggests the following considerations: first, unlike data from hVISA, heteroresistance is widely detected among nonfermenting GNB isolates. In some studies, almost all isolates were found to be heteroresistant even in isolates without prior antibiotic exposure. This first observation highlights that heteroresistance may be an inherent characteristic of these strains. With this in mind, it is difficult to evaluate the impact of this common phenomenon on the clinical practice.

Second, lack of a standard definition of heteroresistance may lead to misidentification of homogeneous strains as heteroresistant, hindering proper assessment of its clinical relevance. Moreover, the best available method to detect in-vitro heteroresistance is the PAP test, but it is a highly time and workforce-consuming method, needs for skilled personnel and performed by reference laboratories. While a standardized detection test is available for hVISA, for nonfermenting GNB detection methods should be further validated.

Third, it is not known whether the presence of in-vitro heteroresistance may lead to in-vivo selection of resistant isolates. Resistant subpopulations with significant fitness cost may be unstable and not sustained over time. Emergence of cefiderocol resistance among CRAB is of clinical concern [62^▪^,^63^]. It is not known if underlying mechanisms are shared between heteroresistant subpopulations and fully resistant isolates. Further studies are needed to describe molecular mechanisms of cefiderocol-heteroresistance among CRAB isolates and to evaluate whether it may be predictive of emergence of resistance during cefiderocol exposure.

Finally, no data demonstrated a relationship between the in-vitro heteroresistance and in-vivo clinical outcome. However, heteroresistance may have clinical implications. It may contribute to treatment failure in particular clinical situations, such as high-inoculum infections or immunocompromised patients. In these cases, antibiotic combination regimens instead of monotherapy may be more appropriate, especially during the first phases of infection.

The presence of heteroresistance among nonfermenting GNB should be translated into clinical practice to prefer combination therapy over monotherapy to prevent the selection of resistant subpopulations during treatment. The use of combination therapy is largely debated in the scientific community. With concern to CRAB, combination regimens are usually used and also suggested by current guidelines in severe infections [64–66]. In an observational study from our group, among patients treated with CRAB infections treated with cefiderocol who experienced microbiological failure, 50% developed resistance to the siderophore antibiotic [58^▪▪^]. The rate of microbiological failure was significantly higher in patients who received monotherapy compared to those who received combination therapy [58^▪▪^]. Thus, awaiting for further studies to better understand the microbiological and clinical role of heteroresistance, caution should be taken for monotherapy among CRAB isolates. Conversely, combination therapy should be preferred when both a colistin-based or cefiderocol-based therapy is chosen. Combination of colistin or cefiderocol with other antibiotics, such as ampicillin/sulbactam, should be preferred for CRAB infections.

In patients with invasive infections caused by *P. aeruginosa*, combination therapy should not be the routine choice but may be considered on a case-by case basis, especially in high-inoculum or in the first phases of infection [65].

## CONCLUSION

Unlike Gram-positive bacteria, heteroresistance is a common in-vitro phenomenon in nonfermenting GNB. It has been studied mainly for *A. baumannii* and *P. aeruginosa* and appears to be frequent for several tested antibiotics, including colistin, tygecicline, cefiderocol, carbapenems. The PAP is the gold standard to detect heteroresistance, but its use has been validated only for hVISA. It is not known if the presence of in-vitro heteroresistance may have a clinical impact on clinical outcome *in vivo*. Studies evaluating this potential correlation are limited and inconclusive.

Since heteroresistance is very common among nonfermenting GNB, it should be considered an inherent characteristic of these strains and cannot be directly predictive of clinical failure. Further studies are needed to better elucidate this point. Unmet needs in this field are represented by the understanding of molecular mechanisms underlying heteroresistance and its role in the potential development of treatment emergent resistance. Of importance, the presence of heteroresistance among nonfermenting GNB should lead to prefer combination regimens instead of monotherapy in the clinical practice, especially in high-inoculum infections.

## Acknowledgements

*None.*

### Financial support and sponsorship

*None.*

### Conflicts of interest

*G.T. received honoraria by Shionogi for educational meetings. M.F. received unconditional grants from MSD and grants/or speaker honoraria from Angelini, Shionogi, Pfizer, Menarini, Gilead, TermoFisher and Nordic Pharma. The remaining authors have no conflicts of interest.*

## REFERENCES AND RECOMMENDED READING

Papers of particular interest, published within the annual period of review, have been highlighted as:

▪ of special interest
▪▪ of outstanding interest
